# Utilization of the litchi male-sterile line ‘MS1’—assisting in the selection of suitable harvesting periods for pollen donors for hybridization

**DOI:** 10.3389/fpls.2026.1685024

**Published:** 2026-01-26

**Authors:** Zhenxuan Quan, Tianjie Deng, Yaoyao Li, Xuewen Zheng, Yi Wang, Weicai Li

**Affiliations:** 1 South Subtropical Crops Research Institute, Chinese Academy of Tropical Agricultural Sciences, Zhanjiang, China; 2 State Key Laboratory of Tropical Crop Breeding, Sanya, China; 3 Key Laboratory of Tropical Fruit Biology, Ministry of Agriculture and Rural Affairs, Zhanjiang, China; 4 Key Laboratory of Postharvest Physiology and Technology of Tropical Horticultural Products of Hainan Province, Zhanjiang, China; 5 Guangdong Engineering Technology Research Center of Tropical Specialty Fruit, Zhanjiang, China; 6 South China Agricultural University, Guangzhou, China

**Keywords:** anther, hybridization, litchi, male-sterile, pollen

## Abstract

Litchi (*Litchi chinensis* Sonn.), an important fruit originating in China, has received increasing attention for improving breeding efficiency in recent years due to the growing adoption of hybridization. It is a monoecious plant and predominantly relies on cross-pollination for seed production. Thus, enhancing pollen quality is essential for fruit set, which is closely associated with the developmental stages of male flowers. In this study, anthers of the litchi cv. ‘D13’ at five late developmental stages (half-exposed stage, fully-exposed stage, filament-elongated stage, anthesis, and browning stage) were investigated using paraffin sectioning, staining, *in vitro* germination, and controlled pollination techniques. Cytological characteristics of pollen were evaluated using paraffin sectioning, while pollen viability was assessed by I_2_-KI staining, and the germination rate was determined through *in vitro* culture on sucrose media. Furthermore, pollen collected from these five stages was used for controlled pollination of the male-sterile line ‘MS1’. The results indicated that pollen collected at the filament-elongated stage and anthesis exhibited higher viability and germination potential, which resulted in improved fruit set in the male-sterile line ‘MS1’. The study identified the filament-elongated stage and anthesis as optimal stages for pollen collection in litchi.

## 1 Introduction

Litchi (*Litchi chinensis* Sonn.), which originated in Yunnan, China, has been cultivated for over 2000 years (Hu et al., 2022). With improvements in living standards, consumer demand for diverse litchi flavors has increased, thereby accelerating litchi breeding efforts. Over the past three decades, more than 38 new litchi varieties have been developed in China, and most were selected from superior plants identified through germplasm resource surveys (Wu et al., 2007; Chen et al., 2019). However, due to the increasing depletion of wild resources, this breeding method exhibits inherent limitations, such as low efficiency and a passive, resource-dependent nature. Artificial hybridization can generate a large pool of breeding resources to compensate for the depletion of wild resources. Despite its increasing adoption in recent years, the technique remains in its infancy, resulting in low efficiency (Xiang et al., 2011). Breeding efficiency is closely associated with multiple factors, including weather conditions, pollination techniques, the nutritional status of trees, and cultivation management (Gupta et al., 2018; Xie et al., 2019). Previous studies have focused on pollen quality at different developmental stages and on the effects of different storage conditions on pollen germination rates (Wang et al., 2015; Wu et al., 2017; Gupta et al., 2018). Nevertheless, these investigations have primarily addressed individual aspects, with limited integration of these factors. Pollen vitality varies across different stages of anther development. Thus, an ideal pollen donor should produce both a high quantity of pollen and high-quality pollen for successful hybridization (Aizen and Harder, 2007; Chu et al., 2015; Wang et al., 2024). Furthermore, maintaining pollen viability is crucial for effective pollination, necessitating appropriate *in vitro* storage conditions (Chai et al., 2023; Shemer et al., 2014). However, current identification of suitable pollen donors still relies primarily on visual assessment of male floral traits, lacking validation through field experiments.

In this study, pollen viability and pollination efficiency were evaluated using pollen collected from the last five developmental stages of male-fertile litchi cv. ‘D13’ to select suitable pollen donors. The recently discovered male-sterile litchi line, named ‘male-sterile line 1’ (‘MS1’) (Quan et al., 2023), was used for field verification. This study aimed to clarify the following key questions: the relationship between pollen viability and anther development, the morphological characteristics of male flowers associated with the highest germination rate, and methods for obtaining pure pollen to enhance breeding efficiency. The objective was to establish a theoretical foundation for litchi hybridization and to optimize production management schedules. The experimental data provide definitive insights into these questions.

## 2 Materials and methods

### 2.1 Materials and sampling

In this study, the litchi male-sterile line ‘MS1’ served as the maternal parent for field verification, and the male-fertile litchi cv. ‘D13’, which has a similar phenological period, served as the pollen donor. ‘MS1’ was introduced from abroad, while ‘D13’ was developed by South China Agricultural University (Ouyang et al., 2009). They were grafted onto ‘Huaizhi’ rootstocks between 2009 and 2017 and are currently maintained in the Litchi Genetic Resource Garden at the South Subtropical Crops Research Institute, Chinese Academy of Tropical Agricultural Sciences. A total of ten ‘MS1’ plants and twenty ‘D13’ plants were used. All plants were healthy and uniform, with stable male-sterile (‘MS1’) and male-fertile (‘D13’) phenotypes, respectively.

The flower spikes of ‘D13’ were sampled between 9:00 and 10:00 a.m. on March 1, 2023, under sunny conditions with an ambient temperature of approximately 22 °C. After sampling, the flower spikes were transported to the laboratory for subsequent use.

### 2.2 Visual assessment

The flowers were separated from the spikes and classified according to their appearance. Photographs were taken using a Sony α77 camera, with a consistent focusing distance (approximately 1.5 cm) and exposure settings to accurately represent the actual size and color.

### 2.3 Paraffin sectioning

The paraffin sectioning process was performed according to the protocols described by Hu et al. (2021). Flowers were fixed using FAA fixation solution (38% formaldehyde: glacial acetic acid: 70% ethanol = 1:1:18, v/v/v) for more than 24 h. The fixed samples were then dehydrated through a graded ethanol series (70%, 80%, 95%, and 100%), each for 15 min. Subsequently, the samples were treated twice with pure xylene, with each incubation lasting 15 min. The flowers were then immersed in a temperature-controlled pure wax pot set at 60 °C for 72 h. Following immersion, the samples were embedded and sectioned to a thickness of 8 µm. Afterward, the wax was dissolved using xylene for 20 min. The sections were stained with 1% safranine solution for 5 min and subsequently dehydrated using graded ethanol solutions (70%, 80%, 95%, and 100%), each for 2 min. Xylene was applied for 10 min to permeabilize the samples. Permanent specimens were prepared using neutral gum, and images were captured using a microscope (80i, Nikon, Japan). The images were edited using Adobe Photoshop 2021.

### 2.4 Assessment of pollen amount, viability, and germination rate

The experiments were conducted with reference to the methods described by Zhang and Liu (2015), Guo et al. (2016), and Cao et al. (2017), with minor modifications. Fresh ‘D13’ male flowers were collected and classified based on their appearance. One hundred flowers (approximately 700 anthers) were collected, placed on dry paper, and dried using a blast dryer at 30 °C for 12 h. After drying, the anthers were gently ground and sieved until no pollen remained underneath. The fallen pollen was collected in a centrifuge tube, and 200 µL of liquid culture medium (10% sucrose, 0.01% boric acid, w/v) was added to prepare the pollen suspension for subsequent use.

#### 2.4.1 Determination of pollen germination rate

The solid culture medium (10% sucrose, 0.01% boric acid, 0.5% agar, w/v) was prepared and uniformly applied onto a clean glass slide, then allowed to cool. A clean culture dish was prepared by lining it with a moist paper towel and placing a rubber pad on top to avoid direct contact between the tissue and the glass slide. A 100 µL aliquot of the pollen suspension was applied to the solid culture medium, and the entire slide was placed into the culture dish. The dish was then covered, sealed with plastic tape, and incubated at 25 °C for 8 h. After incubation, the slide was removed, and microscopic images were captured.

For each developmental stage, five microscopic fields of view with even distribution and minimal overlap were systematically selected for germination assessment. The germination rate was determined by directly counting the number of pollen tubes in each field of view and calculated as follows: number of germinated pollen grains/total number of pollen grains × 100%.

#### 2.4.2 Assessment of pollen amount and pollen viability

A 100 µL aliquot of I_2_-KI solution (0.33% I_2_, 0.66% KI, w/v) was added to the remaining 100 µL pollen suspension for staining. The mixture was gently shaken and immediately examined under a microscope to capture images. The I_2_–KI solution was used both to assess pollen viability and to analyze changes in pollen quantity at different developmental stages, based on the differential staining response whereby immature litchi pollen grains stain dark with I_2_–KI, while mature grains do not.

Pollen grains exhibiting a round shape and bright appearance were classified as viable, whereas shrunken or dark grains were considered immature and non-viable. For each developmental stage, five microscopic fields of view with even distribution and minimal overlap were selected for viability assessment. Pollen viability was calculated as follows: number of unstained pollen grains/total number of pollen grains × 100%.

### 2.5 Determination of stored pollen germination rate

Pollen was collected as described in Section 2.4 and stored at room temperature. Subsequent experiments were carried out to determine the germination rate of the stored pollen after 1, 3, 6, 10, and 15 days. Before analysis, the volume of the collected pollen was estimated, and the samples were mixed with 100 times their volume of liquid culture medium (10% sucrose, 0.01% boric acid, w/v). The samples were thoroughly shaken to obtain a uniform pollen suspension. Germination rate measurements were then conducted following the procedure described in Section 2.4.1.

### 2.6 Hybridization

Pollen was collected as described in Section 2.4, and hybridization was performed according to Zhu et al. (2019). The pollen was dispersed and shaken in 50 mL sucrose solution (1%, w/v) to prepare a pollen suspension after reaching the field. Following filtration through a 200-mesh sieve, each pollen group was sprayed onto five ‘MS1’ female flower spikes of relatively uniform size, ensuring that the stigma had just cracked and that droplets were visible. To prevent mildew, the spikes were bagged after they had slightly dried for a few minutes. This procedure was performed once between 9:00–10:00 a.m. and 3:00–4:00 p.m. during flowering to ensure sufficient pollination of female flowers. The fruit-set number was recorded 30 days after pollination.

### 2.7 Microscopic observation of anther grinding liquid

Fresh litchi flowers were collected and transported to the laboratory, where ten mature flowers (approximately 70 anthers) were removed using tweezers. The anthers were macerated with an appropriate volume of sterile water and 1% aniline safranine solution for 15 min. After staining, the anthers were washed with distilled water and transferred to a mortar, where 1 mL of distilled water was added for grinding. The anther dispersion was then filtered through a 200-mesh sieve, and the resulting liquid was observed under a microscope.

### 2.8 Scanning electron microscope observation

Fresh litchi flowers were harvested and transported to the laboratory. Mature anthers were removed using tweezers and dried in a silica gel dryer for 72 h. The dried anthers were mounted on a copper platform, coated with a conductive adhesive, gold-sputtered, and imaged at an accelerating voltage of 10 kV using a scanning electron microscope (MIRA3 LMU, Tescan, Czech Republic). The images were edited using Adobe Photoshop 2021.

### 2.9 Data analysis

All experiments were conducted in at least three independent trials. Results were expressed as mean ± standard deviation. Data variance analysis was performed using SPSS Statistics 26.0 software (IBM Corporation, Armonk, NY, USA), with *p* < 0.05 considered statistically significant. Figures were plotted using Origin 2024 software (Origin Lab Corporation, Northampton, MA, USA).

## 3 Results

### 3.1 Morphological characteristics of ‘D13’ male anthers during the late developmental stages

**Figure 1:**
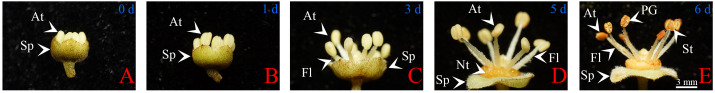
Visual depiction of different stages during the late developmental period of cv. ‘D13’ male flowers. **(A)** Male flower at the anther half-exposed stage. **(B)** Male flower at the anther fully exposed stage. **(C)** Male flower at the filament-elongated stage. **(D)** Male flower at anthesis. **(E)** Male flower at the browning stage. At, anther; Fl, filament; Nt, nectar; PG, pollen grains; Sp, sepal; St, stomium.

Fresh ‘D13’ male flowers were carefully collected and classified based on specific criteria, including the anther half-exposed stage, anther fully exposed stage, filament-elongated stage, anthesis, and browning stage. At the anther half-exposed stage, the anthers were tightly enfolded by the sepals, with approximately half of the anthers protruding externally (**Figure 1A**). As the anthers progressed to the fully exposed stage, they became completely exposed (**Figure 1B**). Immediately afterward, the filaments protruded from the receptacle as the anthers developed to the filament-elongated stage (**Figure 1C**). Subsequently, at anthesis, the sepals were completely open and the filaments reached their maximum length. Anther dehiscence was not common at this stage (**Figure 1D**). When the anthers entered the browning stage, most anthers turned brown and cracks appeared, through which pollen grains were released (**Figure 1E**).

### 3.2 Cytological characteristics of ‘D13’ male anthers during the late developmental stages

**Figure 2:**
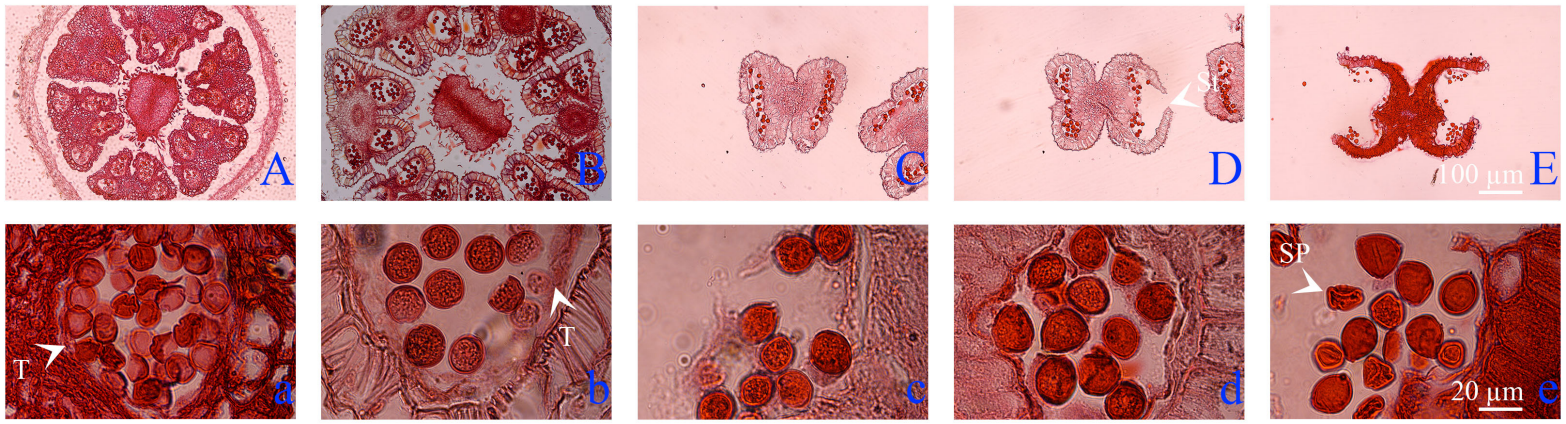
Anther structure of cv. ‘D13’ male flowers. **(A)** Anthers at the half-exposed stage (100×). **(B)** Anthers at the fully exposed stage (100×). **(C)** Filament-elongated anthers (100×). **(D)** Anthers at anthesis (100×). **(E)** Anthers at the browning stage (100×). **(a–e)** Enlarged views of anther chambers at the corresponding five stages (1000×). SP, shrunken pollen grain; St, stomium; T, tapetum.

Paraffin sectioning was performed to observe cytological characteristics of cv. ‘D13’ during the late developmental stages. The results showed that, at the anther half-exposed stage, the anthers were enveloped by sepals and four distinct chambers were observed (**Figure 2A**). Inside the chambers, pollen grains were unformed (**Figure 2a**). When the anthers entered the fully exposed stage, a noticeable increase in size was observed, and fibrous endothecium formation occurred (**Figure 2B**). At this stage, the tapetum was completely degraded and the pollen grains were plump (**Figure 2b**). Subsequently, the anthers developed to the filament-elongated stage, during which the separating tissue between chambers degraded, resulting in bilocular anthers (**Figure 2C**). The pollen grains maintained a consistently round shape (**Figure 2c**). At anthesis, some anthers began to crack and pollen grains were released (**Figures 2D, d**). After flowering, the remaining pollen grains within the chambers were markedly reduced, and some pollen grains appeared shrunken and deformed (**Figures 2E, e**).

### 3.3 Pollen amount of ‘D13’ male anthers during the late developmental stages

**Figure 3:**
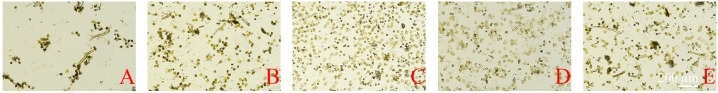
Pollen grains at different stages during the late developmental period of the litchi cv. ‘D13’. **(A)** Pollen grains at the anther half-exposed stage (100×). **(B)** Pollen grains at the anther fully exposed stage (100×). **(C)** Pollen grains at the filament-elongated stage (100×). **(D)** Pollen grains at anthesis (100×). **(E)** Pollen grains at the browning stage (100×).

Microscopic images were acquired, and the average number of pollen grains per field of view was counted. The results indicated that the lowest pollen number (49.2 grains) occurred at the anther half-exposed stage (**Figure 3A**). With further anther development, this number increased to 268 and 495.2 at the fully exposed stage and filament-elongated stage, respectively (**Figures 3B, C**). At anthesis, a substantial number of pollen grains (423.4) was still observed, although this represented a slight decrease compared with the preceding stage (**Figure 3D**). At the browning stage, although the pollen number was significantly reduced (206.6), a considerable amount of pollen remained (**Figure 3E**).

**Figure 4:**
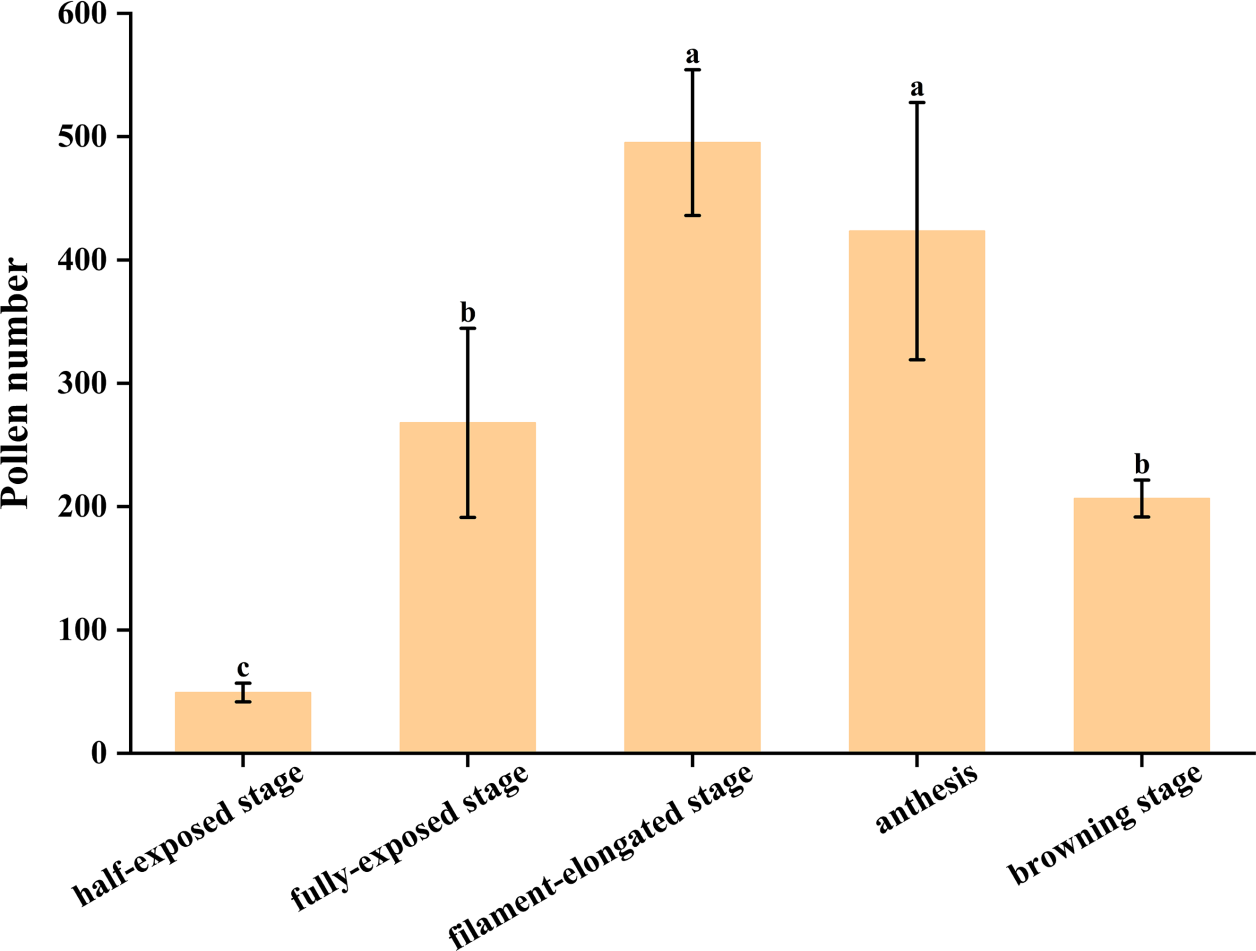
Pollen grain amounts at different developmental stages of cv. ‘D13’ male flowers during the late developmental period (100×). Different letters above the columns indicate significant differences (*p* < 0.05).

Statistical analysis showed an increasing trend in pollen amount from the anther half-exposed stage to the filament-elongated stage, followed by a marked decrease from the filament-elongated stage to the browning stage (**Figure 4**).

### 3.4 Pollen viability and germination rate of ‘D13’ male anthers during the late developmental stages

**Figure 5:**
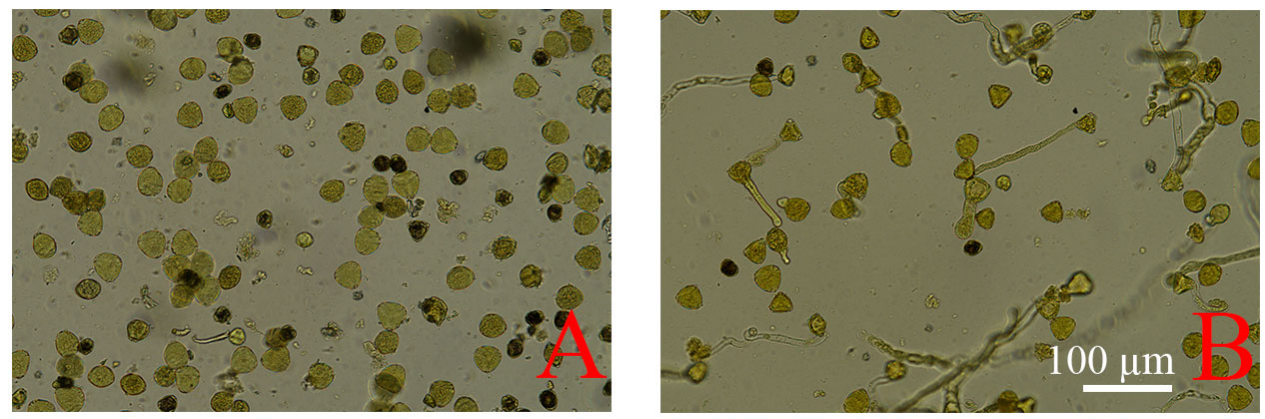
Staining of pollen grains at anthesis of cv. ‘D13’ male flowers. **(A)** Staining of pollen grains at anthesis after 0 h of cultivation (200×). **(B)** Staining of pollen grains at anthesis after 8 h of cultivation (200×).

I_2_–KI solution was applied to assess pollen viability. To distinguish active from inactive pollen, a preliminary staining experiment was conducted. The results showed that immature pollen grains were small in size and stained dark with I_2_–KI; none of these grains germinated after cultivation and were therefore considered inactive. In contrast, active pollen grains were round and plump, some of which germinated after cultivation, and they were not stained by I_2_–KI (**Figure 5**).

**Figure 6:**
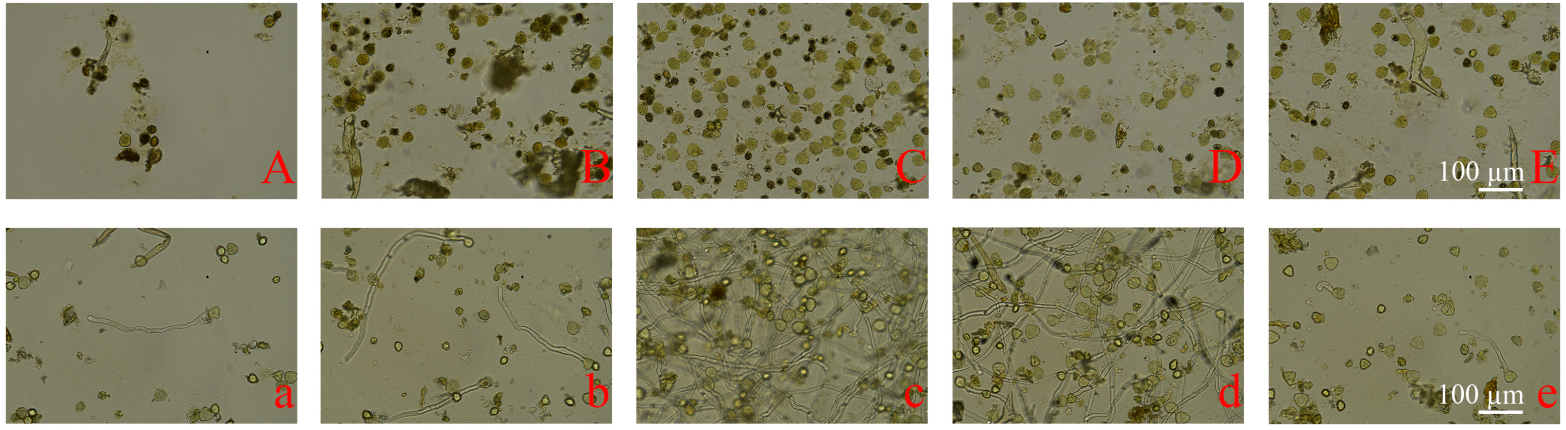
Staining and germination of pollen grains at different stages of male flower development in cv. ‘D13’. **(A)** Staining of pollen grains at the anther half-exposed stage (200×). **(B)** Staining of pollen grains at the anther fully exposed stage (200×). **(C)** Staining of pollen grains at the filament-elongated stage (200×). **(D)** Staining of pollen grains at anthesis (200×). **(E)** Staining of pollen grains at the browning stage (200×). **(a–e)** Germination of pollen grains at the corresponding five stages (200×).

At the anther half-exposed stage, pollen grains were mixed with impurities, and most exhibited dark staining (**Figure 6A**). The average germination rate at this stage was 4.6% (**Figure 6a**). As the anthers entered the fully exposed stage, more pollen grains were observed, still mixed with impurities, and nearly half exhibited dark staining (**Figure 6B**). The germination rate increased to 14.3% compared with the previous stage (**Figure 6b**). At the filament-elongated stage, fewer than one-third of the pollen grains showed dark staining (**Figure 6C**), resulting in a relatively high germination rate (38.6%) and longer pollen tubes (**Figure 6c**). At anthesis, when most pollen grains were clear and transparent (**Figure 6D**), the germination rate was correspondingly high (46.5%; **Figure 6d**). When the anthers entered the browning stage, the pollen germination rate decreased to 8.4%, and more impurities were observed (**Figure 6E**).

**Figure 7:**
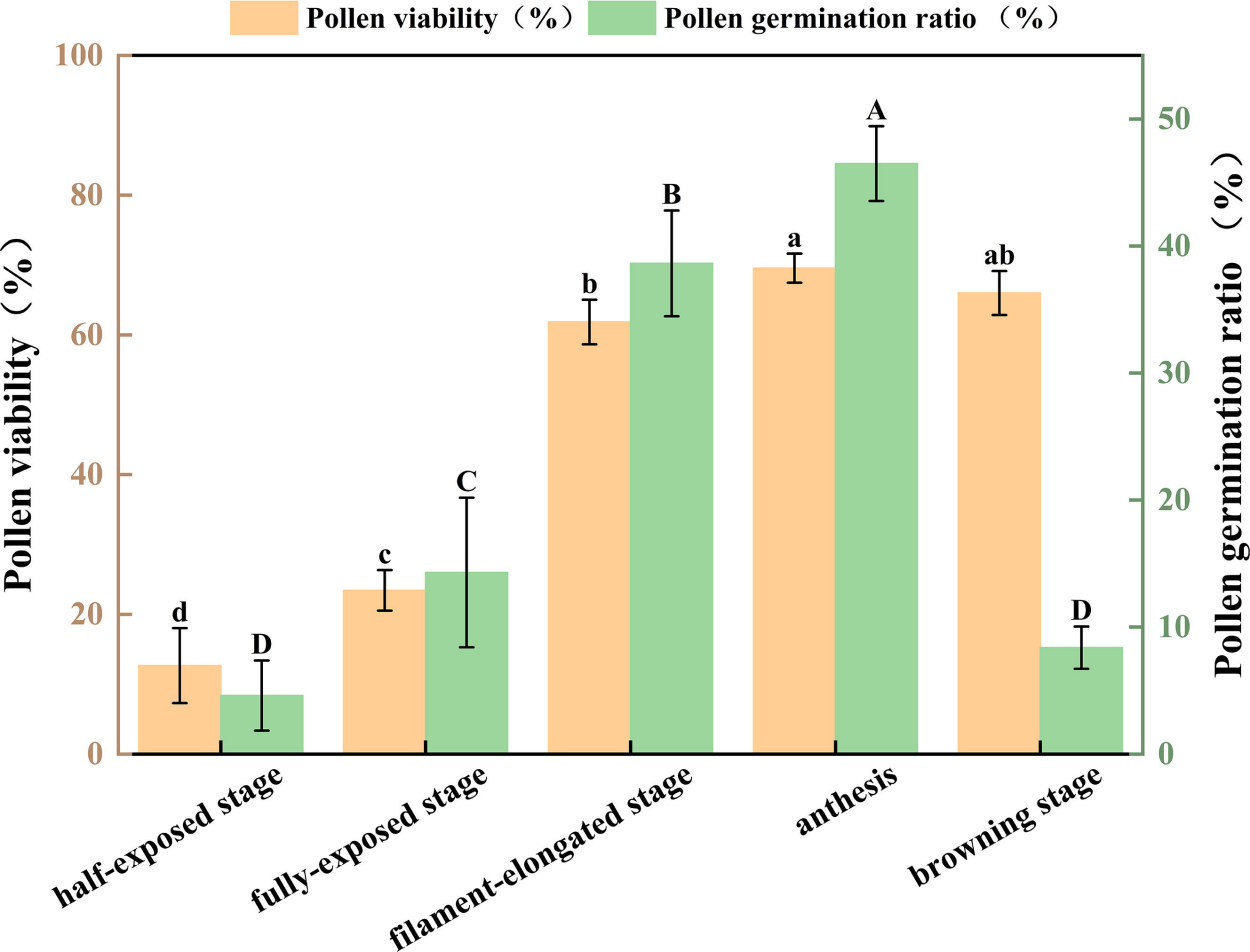
Quantitative analysis of pollen viability and germination rate at late developmental stages of cv. ‘D13’ male flowers. Values represent means of five replicates, with bars indicating the standard error of the mean. Different letters above the columns indicate significant differences (*p* < 0.05).

Statistical analysis indicated that, as anthers progressed from the half-exposed stage to the filament-elongated stage, pollen viability and germination rate increased markedly. In contrast, both parameters declined from the filament-elongated stage to the browning stage. Significant differences in pollen viability and germination rate (*p* ≤ 0.05) were observed among the anther half-exposed stage, anther fully exposed stage, and anthesis (**Figure 7**).

### 3.5 Effect of storage time at room temperature on pollen germination rate of ‘D13’

**Figure 8:**
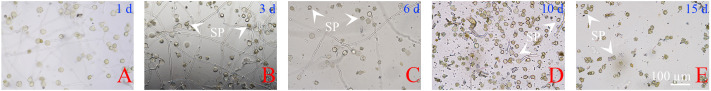
Observations of pollen germination after storage at room temperature. **(A)** Pollen germination after 1-day storage (200×). **(B)** Pollen germination after 3-day storage (200×). **(C)** Pollen germination after 6-day storage (200×). **(D)** Pollen germination after 10-day storage (200×). **(E)** Pollen germination after 15-day storage (200×). SP, shrunken pollen grain.

Male flowers at the filament-elongated stage and anthesis were selected to further evaluate the effect of storage time on pollen due to their high germination rates. The results showed that noticeable pollen grain shrinkage and deformation occurred after 3 days of storage at room temperature (**Figure 8B**). This proportion gradually increased over time, with nearly all pollen grains exhibiting shrinkage and deformation after 15 days of storage (**Figure 8E**).

**Figure 9:**
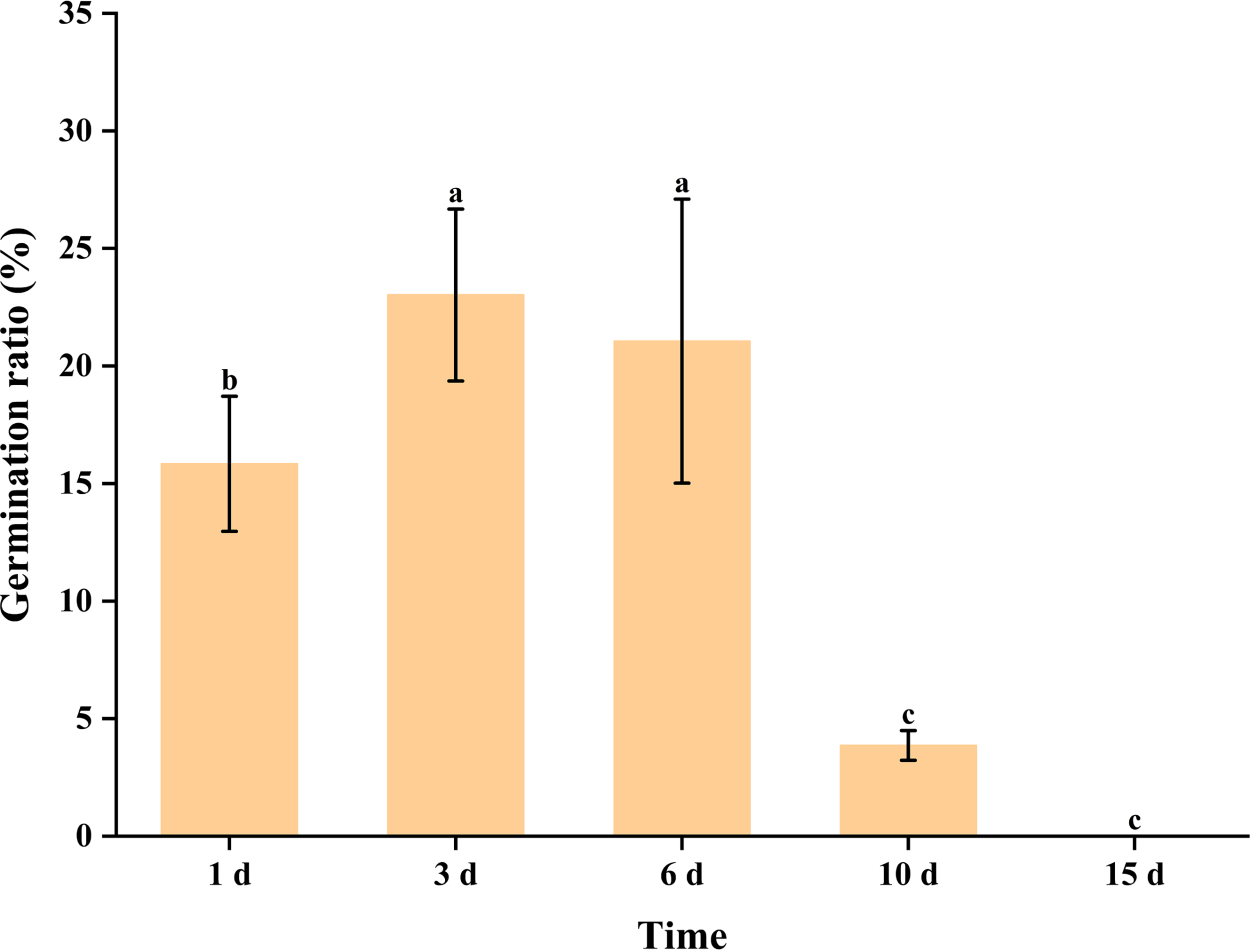
Pollen germination rate after different durations of storage at room temperature. Different letters above the columns indicate significant differences (*p* < 0.05).

Regarding pollen germination rate, relatively high germination was observed within the first 6 days of storage at room temperature. After 6 days, the germination rate remained at approximately 21.1%, but declined to about 3.9% after 10 days and reached zero after 15 days of storage (**Figure 9**).

### 3.6 Verification of male-sterility characteristic in the litchi male-sterile line ‘MS1’

**Figure 10:**
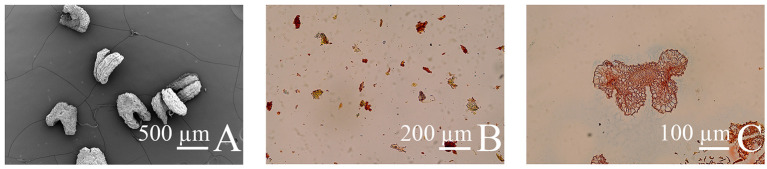
Verification of male-sterility characteristics in the litchi male-sterile line ‘MS1’. **(A)** Scanning electron microscope (SEM) observation of mature male anthers of ‘MS1’ (76×). **(B)** Grinding liquid of anthers from ‘MS1’ (200×). **(C)** Paraffin section of mature male anthers of ‘MS1’ (100×).

Before pollination between ‘D13’ and ‘MS1’, SEM observation, anther grinding, and paraffin sectioning were performed to verify the male-sterile characteristics of ‘MS1’. The results showed that mature anthers of ‘MS1’ were thin and wrinkled (**Figure 10A**), with no pollen grains present (**Figures 10B, C**). These findings confirm that ‘MS1’ is a reliable male-sterile material for hybridization.

### 3.7 Fruit-set number in the hybridization between ‘D13’ and ‘MS1’

**Figure 11:**
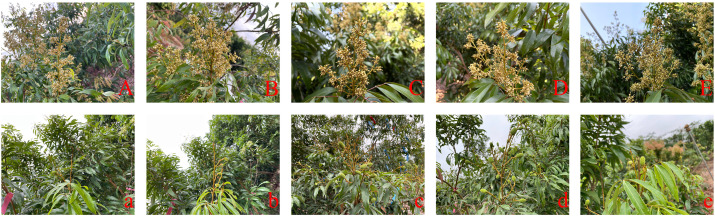
Fruit set of the litchi male-sterile line ‘MS1’ after spraying pollen suspensions from cv. ‘D13’. **(A–E)** Spikes of ‘MS1’ sprayed with ‘D13’ pollen suspensions from the anther half-exposed stage, anther fully exposed stage, filament-elongated stage, anthesis, and browning stage, respectively, at 0 days. **(a–e)** Corresponding spikes of ‘MS1’ 30 days after spraying with pollen suspensions from the same five stages.

**Figure 12:**
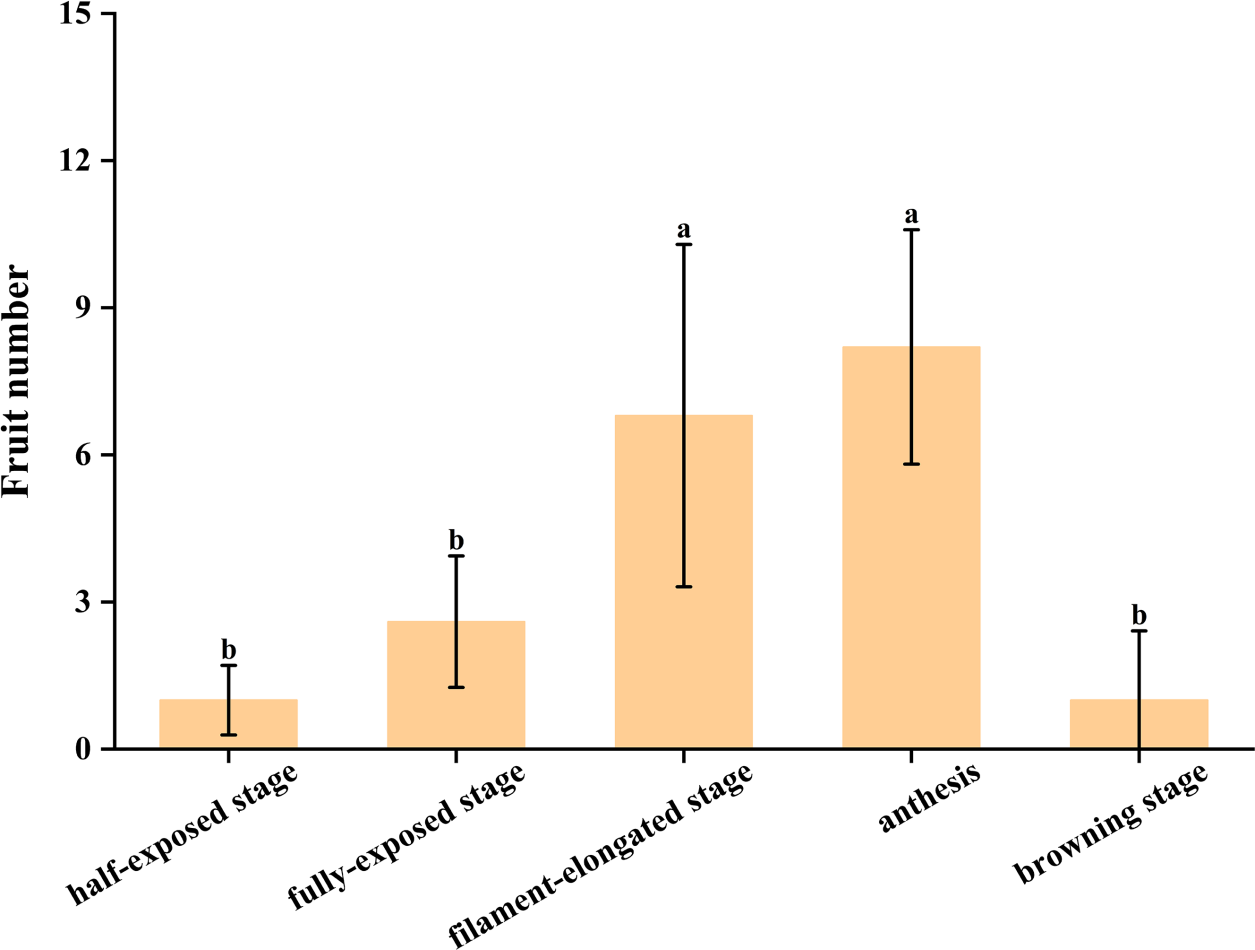
Fruit set resulting from hybridization between cv. ‘D13’ and the male-sterile line ‘MS1’. Different letters above the columns indicate significant differences (*p* < 0.05).

Soon after preparation, the ‘D13’ pollen suspensions were sprayed onto female flower spikes of the litchi male-sterile line ‘MS1’ during anthesis. The fruit-set number was recorded 30 days later. The results indicated that only one fruit per spike, with an 80% fruit set, was obtained on ‘MS1’ after spraying pollen suspension from the anther half-exposed stage (**Figures 11a**; **12**). Compared with the previous stage, an increase was observed (2.6 fruits per spike with 100% fruit set) after application of pollen suspension from the anther fully exposed stage (**Figured 11b**; **12**). Subsequently, when pollen suspension from the filament-elongated stage was applied, a significant increase in fruit-set number (6.8 fruits per spike with 100% fruit set) was observed on ‘MS1’ (**Figures 11c**; **12**). When pollen suspension from ‘D13’ at anthesis was applied, the highest fruit number on ‘MS1’ (8.2 fruits per spike with 100% fruit set) was achieved (**Figures 11d**; **12**). In contrast, application of pollen suspension from the ‘D13’ browning stage resulted in poor fruit-set efficiency (one fruit per spike with 60% fruit set) (**Figures 11e**; **12**).

## 4 Discussion

### 4.1 Pollen maturation is closely related to anther development

The development of male reproductive organs is fundamental to sexual reproduction and crop yield in flowering plants (Ji et al., 2013; Fartyal et al., 2025). The anther, a key structure of these organs, undergoes a series of critical developmental stages—including staminal meristem specification, sporogenous cell differentiation, and pollen maturation—to achieve pollination competence (Ma, 2005). As the carrier of male gametes, pollen plays an indispensable role in successful fertilization, species propagation, and biodiversity maintenance (Du et al., 2025; Lv et al., 2024).

In some plants, such as rice (*Oryza sativa*), maize (*Zea mays*), and *Arabidopsis thaliana*, anther development can be divided into several distinct stages corresponding to three major phases of pollen development: microsporogenesis, post-meiotic microspore development, and microspore mitosis (Chaudhury et al., 2010; Zhang and Wilson, 2009; Han et al., 2022; Sanders et al., 1999). The morphology and function of microspores or pollen grains differ across developmental stages. Abnormally developed pollen may lead to reduced pollination and fertilization efficiency, or even sterility (Pei et al., 2024; Doğan and Vardar, 2023; Hu and Gao, 2022; Lei et al., 2022; Wang et al., 2008; Zhu et al., 2012). In this study, five late developmental stages of litchi anthers were examined, allowing observation of pollen progression from immaturity to maturity and subsequent senescence. During this process, the formation of fibrous endothecium was also observed, which may be associated with mechanical anther dehiscence for pollen release (Alves et al., 2025). This morphological feature can be readily identified and may assist breeders in improving operational efficiency. Our results indicate that the pollen germination rate reached its peak at the filament-elongated stage and anthesis. This finding is consistent with previous reports (Gupta et al., 2018; Wu et al., 2017), while the present study provides a more detailed analysis of earlier anther developmental stages.

### 4.2 Pollen germination is an expression of pollen viability under certain conditions

Pollen viability determined by staining methods is based on the reaction between staining solutions and intracellular substances and is generally faster than culture-based methods. However, pollen viability assessed by staining was higher than the *in vitro* germination rate. This trend is consistent with previous findings in tobacco, cucumber, tomato, and pepper (Heidmann et al., 2016). Although some pollen grains were identified as viable by staining, their capacity for effective germination remained limited. This discrepancy may be attributed to differences in optimal germination conditions required by individual pollen grains, which could not be fully established in the present study.

Here, the I_2_–KI staining method was used to determine pollen viability based on starch accumulation in pollen grains. Theoretically, starch accumulation is often higher in mature pollen of some plants (such as mango and wheat), so the dark pollen grain stained by I_2_-KI solution is considered to be viable (Ma et al., 2011; Li et al., 2013). However, the substances accumulated in the mature pollen of some plants are lipids, such as *Camellia oleifera*. The lipid-accumulated pollen cannot be stained by I_2_-KI, which is similar to our observations and suggests that mature litchi pollen may be lipid-type. Generally, lipid-type pollen accumulates starch during the bicellular stage, which is later degraded (Wei D. et al., 2013). At the anther half-exposed, fully exposed, and filament-elongated stages, some pollen grains stained with I_2_–KI may therefore represent pollen at the bicellular stage. Consequently, pollen germination rate, rather than staining-based viability, is a more reliable indicator for evaluating pollen availability in litchi breeding. Future studies should screen staining methods that more accurately reflect actual litchi pollen viability.

Previous research has shown that cultivars exhibiting higher *in vitro* pollen germination percentages also tend to achieve higher fruit set under field conditions (Pereira et al., 2014; Neto et al., 2009). After collection, successful fertilization by mature pollen depends on appropriate storage conditions and favorable environments prior to contact with the receptive stigma. Pollen purity is critical, as pollen germination is significantly inhibited when excessive impurities are present in the culture medium (Weng et al., 1990). In addition, dehydration, incubation temperature, and incubation duration are key factors influencing pollen germination. For example, Wang et al. (2015) observed no significant difference in pollen germination after dehydration at either 30 °C or 35 °C for 7 hours, suggesting that germination is tolerant of dehydration within this temperature range. For *in vitro* germination, a temperature range of 25-28 °C has been commonly employed by researchers in China (Zhang and Liu, 2015; Li et al., 2016). Meanwhile, studies in India have identified 30 °C as optimal for local litchi cultivars (Gupta et al., 2018), potentially reflecting climatic adaptation within the same species across regions. Furthermore, multiple studies have shown that adding low concentrations of sucrose and boric acid to the culture medium significantly enhances the pollen germination rate (Fu, 2001; Zhang and Liu, 2015; Gupta et al., 2018). Consequently, similar medium formulations have been widely applied *in* pollen culture and hybridization. In the present study, these established methods were adopted and yielded satisfactory results.

Phenological differences also exist among litchi cultivars. Early- and late-maturing cultivars often do not overlap in flowering time, making artificial pollination particularly important. However, pollen has a short lifespan (Dafni and Firmage, 2000; Ren et al., 2019), and flowering asynchrony between parental plants poses challenges for successful hybridization (Jia et al., 2022). Wang et al. (2015) reported that litchi pollen maintained a germination rate exceeding 70% after two years of storage at −86 °C, whereas germination declined to below 5% after 16 days at 25 °C. Similarly, Xiang et al. (1994) observed a complete loss of germination after one year of storage at room temperature. However, data on germination rates following short-term room-temperature storage (<16 days) are limited. In this study, litchi pollen stored at room temperature (16–25 °C) for up to 6 days maintained a relatively high germination rate. These findings indicate that litchi pollen remains viable after short-term room-temperature storage and provide practical guidance for breeders aiming to reduce storage costs.

### 4.3 Future research and applications of the litchi male-sterile line ‘MS1’

In recent years, researchers have extensively investigated the quality and developmental periods of litchi pollen (Wang et al., 2015; Wu et al., 2017; Li et al., 2016). However, most studies have focused on isolated factors, such as germination rate and incubation temperature. Few studies have established a clear relationship between pollen viability and anther development, indicating the need for further investigation.

The present study provides a comprehensive analysis of late-stage pollen development by jointly assessing morphology, staining response, and germination rate. This integrated approach enables a more accurate determination of anther developmental stages in litchi male flowers. Although previous studies have examined phenological growth and floral developmental stages in litchi (Wei Y. et al., 2013; Song, 2021), these analyses remain incomplete. Future work will integrate established models of anther development from other plant species to elucidate the complete developmental process of litchi anthers (Browne et al., 2018; Hu et al., 2022; Zhang and Wilson, 2009). This endeavor will provide valuable insights for breeding programs and for the identification of male-sterile lines.

Furthermore, although previous research has demonstrated that *in vitro* pollen germination rate is directly proportional to fruit set in the field when the same pollen is used for pollination (Pereira et al., 2014), verification of this relationship has been challenging due to potential pollen contamination from male flowers on the same tree. Recently, a pollen-free litchi male-sterile line, ‘MS1’, was identified (Quan et al., 2023). In the present study, this line was used to eliminate pollen contamination and to evaluate the effect of pollen quality on fruit set under field conditions. A clear correlation was observed, thereby confirming this relationship.

Male sterility, defined as the inability of a plant to produce viable pollen, is widely exploited in plant breeding (Brownfield, 2021). Like other crops, the litchi male-sterile line ‘MS1’ exhibits several distinctive traits, including a strong capacity for parthenocarpy, enabling the production of seedless fruits, as observed in crops such as Satsuma mandarin (*Citrus unshiu*) (Jiang et al., 2023) and pineapple (*Ananas comosus*) (Zhang, 2020), which are favored in the consumer market (Chen et al., 2021). In addition, heterosis was observed in some first filial generation plants, which exhibited more vigorous growth and larger fruits compared with the litchi male-sterile line ‘MS1’, consistent with breeding expectations. These superior plants represent a valuable foundation for varietal improvement. In addition, we also found that some plants in the first generation of the litchi male-sterile line ‘MS1’ exhibit leathery leaves (not reported in litchi), which may be related to the local adaptive evolution strat of ‘MS1’ and warrants further investigation.

## 5 Conclusions

**Figure 13:**
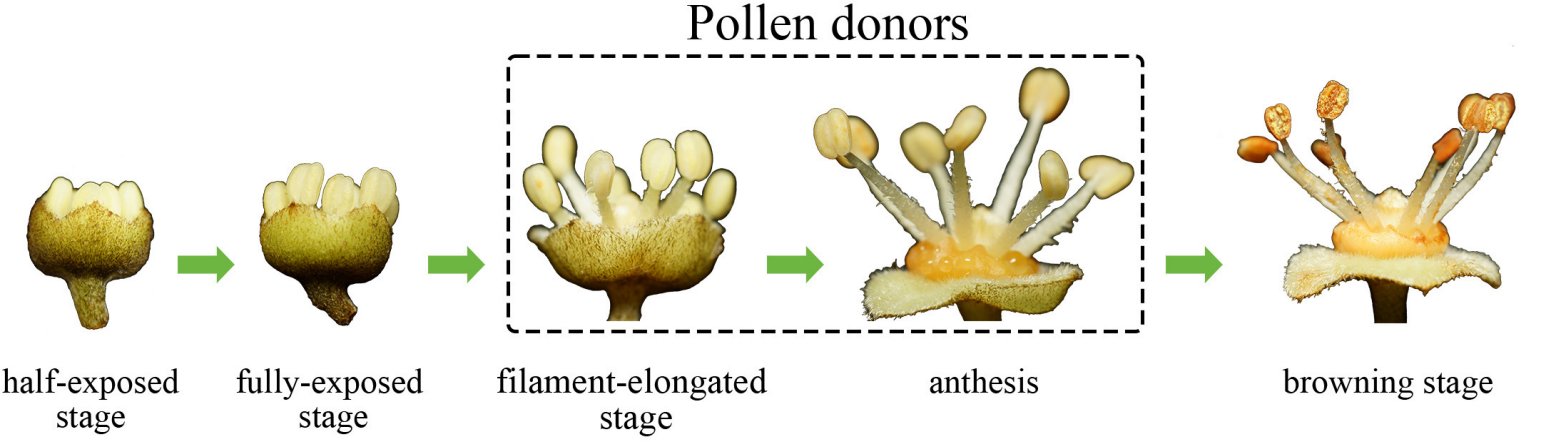
Schematic diagram of male flower developmental stages suitable for use as pollen donors in litchi hybridization.

In this study, pollen amount, pollen viability, germination rate and fruit-set number after pollination of the last five anther developmental stages, it was identified that the male flowers in filament-elongated stage and anthesis were eligible to be chosen as pollen donors. These two stages exhibited favorable characteristics, including higher pollen amounts and higher pollen germination rates, which are associated with increased hybridization efficiency in litchi (**Table 1**; **Figure 13**). In addition, storage experiments showed that fresh litchi pollen should preferably be used within 6 days after collection to maintain an adequate germination rate.

**Table 1:** Description of male flowers in cv. ‘D13’ at different developmental stages.

Stages of anthers	Ease of anther separation	Pollen yield (per view)	Pollen viability (%)	Germination rate (%)	Fruit-set number	Potential suitability as pollen donors
Half-exposed	Hard	49.2 ± 7.56 c	12.7 ± 5.36 d	15.8 ± 2.88 b	1.0 ± 0.71 b	No
Fully-exposed	Easy	268.0 ± 76.68 b	23.4 ± 2.90 c	23.0 ± 3.65 a	2.6 ± 1.34 b	No
Filament-elongated	Easy	495.2 ± 59.06 a	61.8 ± 3.19 b	21.0 ± 6.04 a	6.8 ± 3.49 a	Yes
Anthesis	Easy	423.4 ± 104.36 a	69.5 ± 2.08 a	3.68 ± 0.63 c	8.2 ± 2.39 a	Yes
browning	Hard	206.6 ± 14.98 b	66.0 ± 3.15 ab	0.0 ± 0.00 c	1.0 ± 1.41 b	No

different letters in the same column indicate significant differences (*p* < 0.05).

Overall, this study provides a convenient, rapid, and efficient framework for artificial hybridization in litchi, encompassing pollen collection, preservation, and application, thereby effectively accelerating the litchi breeding process.

## Data Availability

The original contributions presented in the study are included in the article/**Supplementary Material**. Further inquiries can be directed to the corresponding authors.
